# Identification of Dual Receptor Binding Protein Systems in Lactococcal 936 Group Phages

**DOI:** 10.3390/v10120668

**Published:** 2018-11-27

**Authors:** Stephen Hayes, Yoan Duhoo, Horst Neve, James Murphy, Jean-Paul Noben, Charles M. A. P. Franz, Christian Cambillau, Jennifer Mahony, Arjen Nauta, Douwe van Sinderen

**Affiliations:** 1 School of Microbiology & APC Microbiome Ireland, University College Cork, Western Road, Cork T12 YT20, Ireland; stephen.hayes@umail.ucc.ie (S.H.); james.murphy@umail.ucc.ie (J.M.); j.mahony@ucc.ie (J.M.); 2 Architecture et Fonction des Macromolécules Biologiques, Centre National de la Recherche Scientifique (CNRS), Campus de Luminy, 13288 Marseille, France; Yoan.Duhoo@afmb.univ-mrs.fr (Y.D.); cambillau@afmb.univ-mrs.fr (C.C.); 3 Department of Microbiology and Biotechnology, Max Rubner-Institut, 24103 Kiel, Germany; horst.neve@mri.bund.de (H.N.); charles.franz@mri.bund.de (C.M.A.P.F.); 4 Biomedical Research Institute, Hasselt University, 3500 Diepenbeek, Belgium; jeanpaul.noben@uhasselt.be; 5 Architecture et Fonction des Macromolécules Biologiques, Aix-Marseille Université, Campus de Luminy, 13288 Marseille, France; 6 FrieslandCampina, 3818 Amersfoort, The Netherlands; arjen.nauta@frieslandcampina.com

**Keywords:** virus, lactic acid bacteria, structure, host interactions

## Abstract

*Siphoviridae* of the lactococcal 936 group are the most commonly encountered bacteriophages in the dairy processing environment. The 936 group phages possess a discrete baseplate at the tip of their tail—a complex harbouring the Receptor Binding Protein (RBP) which is responsible for host recognition and attachment. The baseplate-encoding region is highly conserved amongst 936 phages, with 112 of 115 publicly available phages exhibiting complete synteny. Here, we detail the three exceptions (Phi4.2, Phi4R15L, and Phi4R16L), which differ from this genomic architecture in possessing an apparent second RBP-encoding gene upstream of the “classical” *rbp* gene. The newly identified RBP possesses an elongated neck region relative to currently defined 936 phage RBPs and is genetically distinct from defined 936 group RBPs. Through detailed characterisation of the representative phage Phi4.2 using a wide range of complementary techniques, we demonstrated that the above-mentioned three phages possess a complex and atypical baseplate structure. Furthermore, the presence of both RBPs in the tail tip of the mature virion was confirmed, while the anticipated host-binding capabilities of both proteins were also verified.

## 1. Introduction

Owing to the potentially adverse economic impact phages have on commercial food fermentations, phages of lactic acid bacteria have become one of the most intensely studied groups of viruses [1]. Phages of *Lactococcus lactis* can be divided into ten groups, incorporating members of both the *Podoviridae* and *Siphoviridae* families [2]. Members of three of these phage groups are most commonly encountered in the dairy processing environment: the lytic c2 and 936 groups, and the P335 phage group, which possesses both lytic and temperate members. Among these, the 936 group phages appear to be the most prevalent [3] with 115 full genome sequences of 936 phages available as of January 2018 [4]. The 936 phages follow a strictly lytic lifestyle and possess a double-stranded DNA genome encapsulated in an isometric capsid (45–60 nm). They also exhibit a long, non-contractile tail of 130–165 nm in length [5].

The initial stage of phage infection involves a very specific interaction between the phage Receptor Binding Protein (RBP), located at the distal end of the tail, and the receptor located at the surface of the bacterial host cell [6]. During the last decade, phage–host adsorption mechanisms of a diverse range of phages have been characterised to an extraordinary level of detail [7,8], including those of *Escherichia coli* phage T4 [9,10], *Bacillus subtilis* phage SPP1 [11,12], and a number of lactococcal phages [13,14,15,16,17]. Despite the enormous diversity of the *Siphoviridae* family, which currently (as of 2017, International Committee on Taxonomy of Viruses) encompasses 133 genera and hundreds of species of phages infecting a diverse range of bacterial and archaeal hosts, the tail architecture of these phages is rather conserved [18]. Phage tails consist of a hollow stack of dozens of homohexameric Major Tail Protein (MTP) rings, with the tail Tape Measure Protein (TMP) filling the length of the otherwise hollow tail core, while at the same time determining tail length [19]. The distal end of the tail is capped by a tail adsorption apparatus, which, for phages infecting Gram-positive bacteria such as *L. lactis*, is termed the tail tip or baseplate [15].

The host adsorption apparatus of two lactococcal phages, p2 (a 936 group phage) and TP901-1 (a P335 group phage), have been studied in great detail [14,16,20,21]. The baseplate of TP901-1 is composed of a central hexameric ring of Dit proteins, with a trimer of BppU (upper baseplate protein) bound to each Dit, and a trimer of Receptor Binding Proteins (or lower baseplate protein, BppL) in turn attached to each of these [19]. The baseplate complex is maintained in an “infection ready” state, with RBPs orientated downward towards potential hosts [16]. It is thought that, upon host recognition by the RBPs, a “firing signal” is sent along the phage tail, triggering ejection of the phage genome into the host cell [22].

The baseplate of p2, in contrast, is normally in a “closed” state, with the RBPs orientated upwards, and requires the presence of Ca^2+^ to prompt a conformational change to orientate its RBPs downward in the “open” conformation to facilitate host binding [13]. The characterisation of the baseplate of phage p2 revealed that the complex is composed of three proteins: the Dit (Distal tail protein), the Tal (Tail associated lysin), and the RBP. The ~1 MDa complex was deduced to consist of a central Dit hexameric ring, with a trimer of RBP proteins attached to each Dit. A Tal protein trimer “plugs” the central cavity of the Dit ring, and a second Dit hexameric ring, rotated 180° and sitting on top of the first, completes the baseplate complex [13].

The RBP structures and functional characteristics of two members (p2 and bIL170) of the 936 group have now been studied in detail [14,23,24], with resolved (p2) or partially resolved (bIL170) structures available, and conserved and distinctive features identified. One of the key findings has been the discovery of a conserved modularity between 936 RBPs, with all exhibiting so-called head, neck, and shoulder domains in their structures. In the p2 RBP, the shoulder domain is the region which attaches the RBP to the Dit, and comprises a β-sandwich fold assembling two four-stranded anti-parallel β-sheets [14], with a long helix domain in each RBP allowing it to associate in trimeric form with two additional RBPs. The neck domain is formed by a triple-stranded β-helix organised into four β-strands, resulting in a rigid structure [25]. The RBP head domain, responsible for binding to phage receptors on the host cell, forms a β-barrel, comprised of seven anti-parallel β-strands. Previous studies have demonstrated that 936 group RBPs can be divided into at least five groups based on nucleotide sequence and host [5,26].

The genomic architecture of the baseplate of p2 is highly conserved among the 936 phage group. Of the 115 publicly available sequences of 936 group phages, 112 possess this same conserved gene order, consisting of the *dit* gene, followed by the *tal* gene, a small gene encoding a product of unknown function (*hp*), and the *rbp* gene. However, three phages that deviate slightly in the baseplate-encoding region were isolated from a Dutch Gouda-producing dairy facility [27]. These phages (Phi4.2, Phi4R15L, and Phi4R16L) appear to encode two distinct RBP genes: a “classical” RBP (*rbp2*), which aligns with Group I (based on predicted head-domain structure) RBPs, and an elongated RBP (*rbp1*) that differs significantly to previously described 936 phage RBPs [5,26].

In the present study, a detailed characterisation of the host interactions of Phi4.2 was undertaken. This phage was selected as a representative phage of this dual-RBP phenomenon. Through the application of a range of complementary molecular techniques, including mass spectrometry, electron microscopy (EM), and single gene and “block” cloning strategies, the functionality of these putative RBPs was investigated.

## 2. Materials and Methods

### 2.1. Host Strains and Phages

Strain *L. lactis* 4, isolated from a Dutch dairy starter culture in 2009 [27], was used as the bacterial host. The strain was grown in M17 broth (Oxoid, Basingstoke, UK) supplemented with 0.5% lactose at 30 °C overnight. Phage Phi4.2, isolated from a dairy facility in 2013 [5], was selected as a representative of the dual-RBP phages (Phi4R15L and Phi4R16L were isolated from the same facility at a later time [4]). Phi4.2 was propagated by inoculating a log-phase culture of *L. lactis* 4 supplemented with 10 mM CaCl_2_. Phage lysates were filtered using a 0.45 µm filter (Sartorius, Dublin, Ireland) and stored at 4 °C. Phage p2, used to represent a typical 936 group phage, was propagated on *L. lactis* NZ9000 and stored in the same manner.

### 2.2. Mass Spectrometry Analysis

To determine if both RBP proteins were produced and incorporated into the mature phage, intact phage particles were analysed by mass spectrometry. Phages were first concentrated and purified by a discontinuous caesium chloride (CsCl) density gradient [28]. The proteinaceous material from 50 µL of purified phage (representing approximately 10^11^ Plaque Forming Units (PFU) per mL) was concentrated by chloroform/methanol precipitation as performed previously [29]. The sample was applied to 12% SDS-PAGE and the resulting protein profiles were visualized following staining with 0.25% Coomassie Brilliant Blue (Bio-Rad, Hertfordshire, UK). Protein bands derived from SDS-PAGE analysis were excised, digested with trypsin, and analysed by electrospray ionization–tandem mass spectrometry (ESI-MS/MS) performed as described previously [30,31].

### 2.3. Electron Microscopy

Phi4.2 virions were first concentrated by precipitation with polyethylene glycol 8000 (PEG8000, 10% *w*/*v* final concentration) and purified using two consecutive CsCl density gradient centrifugations; the first gradient at 82,000× *g* for 2.5 h, the second at 340,000× *g* for 18 h, as described previously [28]. Staining was performed with 2% (*w*/*v*) uranyl acetate on freshly prepared ultrathin carbon films. Grids were analysed in a Tecnai 10 transmission electron microscope (FEI Thermo Fisher Scientific, Eindhoven, The Netherlands) at an acceleration voltage of 80 kV. Micrographs were taken with a MegaView G2 charge-coupled device camera (Emsis, Münster, Germany) [32]. Phage p2 was also prepared and imaged in this manner for comparative purposes.

### 2.4. Individual RBP Cloning and Production

*RBP1_Phi4.2_* (ORF20) and *RBP2_Phi4.2_* (ORF21) were individually cloned into pNZ8048 [33]. The primers used to amplify these genes are listed in Supplementary Table S1. Following overnight double digestion of the plasmids and amplicons with relevant restriction endonucleases, overnight ligation (T4 DNA ligase, NEB, Ipswich, MA, USA) was performed to generate pNZ8048_RBP1 and pNZ8048_RBP2. The resulting ligation mixture was introduced into *L. lactis* NZ9000 by electroporation and transformants were selected on M17 agar plates supplemented with 0.5% glucose (GM17) and 10 μg/mL chloramphenicol (Sigma Aldrich, Wicklow, Ireland) at 30 °C. Sanger sequencing was employed to verify the integrity of the generated recombinant constructs (sequencing service provided by MWG Eurofins, Cologne, Germany). Heterologous gene expression using *L. lactis* NZ9000 harbouring pNZ8048_RBP1 or pNZ8048_RBP2 was performed using the Nisin Controlled Expression (NICE) system incorporating a hexahistidine (His) tag for purification as described previously [17,34]. Protein purification of His-tagged RBP1_Phi4.2_ and RBP2_Phi4.2_ using a Ni-NTA agarose column (Qiagen, Manchester, UK) was performed as described previously [17]. The protein concentration of each fraction was quantified using the standard BioRad protein assay (BioRad, Dublin, Ireland) [35]. In the case of RBP1_Phi4.2_, expression was also attempted in the pTX8048 vector [34] in *L. lactis* NZ9000, and the *E. coli* expression vectors pQE30 (Qiagen, Manchester, UK), and pETM11 and pETM30 [36] in *E. coli* BL21(DE3). Finally, two constructs, with RBP1_Phi4.2_ and RBP2_Phi4.2_ fused to a C-terminal fluorescent mCherry tag, were created using the NZYtech Easy Cloning Kit (NZYtech, Lisbon, Portugal) as per the manufacturer’s instructions.

### 2.5. Block Cloning and Expression of Baseplate Proteins

**Figure 1:**
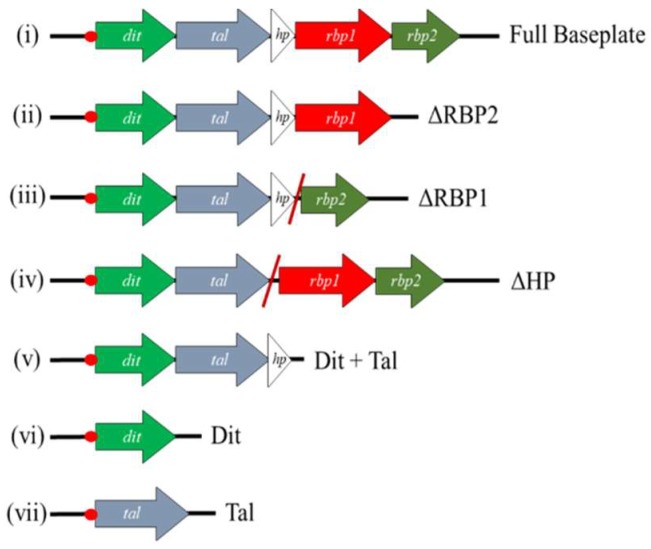
Schematic view of the constructs generated in the pETM11 vector in this study. Diagonal red lines indicate locations where genes have been removed, and adjoining sequence joined together via SOEing PCR. In all cases, a sequence corresponding to a 6× His-tag is incorporated at the 5′-end of the first gene present on the inserted DNA fragment (illustrated by the red dot).

Various DNA fragments each encoding a different combination of baseplate component-encoding genes were cloned into pETM11 [36]. Baseplate constructs incorporating gene deletions were constructed via SOEing PCR as described previously [37], with primers outlined in Supplementary Table S1. The resulting vectors were introduced into *E. coli* BL21(DE3) by heat shock and transformants were selected on Luria–Bertani (LB) agar plates supplemented with 10 μg/mL kanamycin (Sigma Aldrich, Wicklow, Ireland) at 37 °C. The constructs generated using this approach are outlined in Figure 1. Protein expression was performed by adding a 1% inoculum of culture to 800 mL of LB broth and incubating at 37 °C until an OD_600nm_ of 0.8 was reached. Targeted protein expression was induced by the addition of a final concentration of 1 mM IPTG and the culture was then incubated overnight at 20 °C. Cells were harvested by centrifugation at 4000× *g* for 30 min. Pellets were resuspended in 20 mL of lysis buffer (50 mM Tris pH 8.0, 500 mM NaCl, 5% glycerol, 1% triton-X100, 30 mM imidazole, 50 mg lysozyme), and incubated shaking at 4 °C for 30 min, followed by centrifugation at 20,000× *g* for 30 min. Protein was then purified using a Ni-NTA agarose column (Qiagen, Manchester, UK) as described previously [17]. The stability of successfully expressed and purified proteins/protein complexes was assessed via gel filtration using a Superose 6 Increase 10/300 GL column on the AKTA Pure HPLC system (GE Healthcare Life Sciences, Cork, Ireland).

### 2.6. Antibody Production, Western Blot Analysis, and Immunogold Labelling

Polyclonal antibodies were raised in chickens against RBP2_Phi4.2_ from purified protein, and against RBP1_Phi4.2_ using gel slices corresponding to the molecular weight of the RBP1_Phi4.2_ protein when running the entire baseplate complex on a 12% SDS-PAGE gel, by Davids Biotechnologie (Regensburg, Germany) using their standard protocol. Western blot analysis of both RBPs was performed using purified and concentrated phages as described in Section 2.2. Immunogold electron microscopy was performed as described previously with both antibodies, using 5 nm gold conjugates (Sigma-Aldrich, Wicklow, Ireland) in the case of RBP2_Phi4.2_, and 10 nm gold conjugates in the case of RBP1_Phi4.2_ [38].

### 2.7. Adsorption Inhibition Assays

The binding capabilities of the two RBPs were investigated via adsorption inhibition assays as described previously [17]. The RBP-mediated adsorption inhibition assay is an adaptation of the adsorption assay method [39]. Briefly, late exponential phase *L. lactis* 4 (225 μL) cells resuspended in ¼-strength Ringer’s solution were added to a tube to which either 50 μL of protein buffer (negative control), purified RBP2_Phi4.2_ protein (at concentrations of 1.6, 0.5, and 0.1 mg/mL), purified ΔRBP1_Phi4.2_ baseplate (at concentrations of 3.25, 1.62, and 0.8 mg/mL), or the ΔRBP2_Phi4.2_ baseplate (at a concentration of 0.55 mg/mL) was added. This cell–RBP mixture was incubated at 30 °C for 1 h, at which point phages were added at a final concentration of 10^6^ PFU mL^−1^ in a total reaction volume of 0.5 mL (giving an approximate MOI, or Multiplicity of Infection, of 0.01). This mixture was incubated at 30 °C for 12.5 min, before host cells were removed by centrifugation. The supernatant was retained, and the residual titre of remaining phages determined using the double-agar plaque assay method [40]. The control was used to determine base-line adsorption, which was calculated by subtracting residual titre from the initial titre. The resulting figure was then divided by the initial titre to give the adsorption percentage. Adsorption inhibition was calculated as follows: (control adsorption per cent − adsorption per cent after incubation)/control adsorption per cent.

### 2.8. Fluorescent Binding of RBP1_Phi4.2_

Fluorescent binding assays using fluorescently tagged RBP1_Phi4.2_ and RBP2_Phi4.2_ were performed as described previously [41]. Briefly, 0.3 mL of *L. lactis* 4 in the exponential growth phase (taken as an OD_600nm_ of between 0.4 and 0.6) was harvested and resuspended in 100 µL of SM buffer (50 mM Tris-HCl pH 7.5, 100 mM NaCl, 10 mM MgSO_4_). Cells were incubated with either 5 µg/mL of mCherry-RBP1_Phi4.2_ (the maximum quantity possible due to poor expression) or 50 µg/mL of mCherry-RBP2_Phi4.2_ for 12.5 min at 30 °C. Cells were washed twice in SM buffer, and fluorescent binding was visualized by confocal microscopy (Zeiss LSM 5 Exciter, Zeiss, Germany) to achieve high-resolution images, with a wavelength of 514 nm for mCherry. Strain *L. lactis* 10, isolated from the same facility as *L. lactis* 4 [27], was used as a negative control. Images were analysed and compiled using the Zen 2.3 Lite software package (Zeiss, Oberkochen, Germany).

### 2.9. SEC MALS Analysis to Determine Baseplate Size

Size exclusion chromatography was carried out on an Alliance 2695 HPLC system (Waters, Dublin, Ireland) using a Superose 6 HR10/30 column (GE Healthcare, Cork, Ireland) run in a buffer containing 10 mM Tris-HCl, 300 mM NaCl and 50 mL CaCl_2_ at pH 7.5 with a flow rate of 0.6 mL/min. Detection was performed using a three-detector static light-scattering apparatus (MiniDAWN TREOS, Wyatt Technology, Haverhill, UK), a quasielastic light-scattering instrument (Dynapro, Wyatt Technology, Haverhill, UK) and a refractometer (OptilabrEX, Wyatt Technology, Haverhill, UK). Molecular weight calculations were performed with the ASTRA V software (Wyatt Technology, Haverhill, UK) as previously described [42]. Proteins were injected at a final concentration of 1 mg/mL (±0.1 mg/mL). Errors were assigned by the Astra software (Wyatt).

### 2.10. Negative Stain EM Analysis of Baseplate Complexes

For negative staining, 6 μL of each successfully expressed baseplate complex was applied onto glow-discharged carbon-coated grids (Agar Scientific, Stansted, UK) at a concentration of 0.03 mg/mL. The grids were washed three times with 10 μL of deionized water before incubating for 45 s in 1% (*w*/*v*) uranyl formate (Agar Scientific, Stansted, UK). CCD images were collected using a Tecnai Spirit operated at 120 KV and a 2 K × 2 K CCD camera.

## 3. Results

### 3.1. RBP1_Phi4.2_ Is Distinct from Current 936 RBP Groups

**Figure 2:**
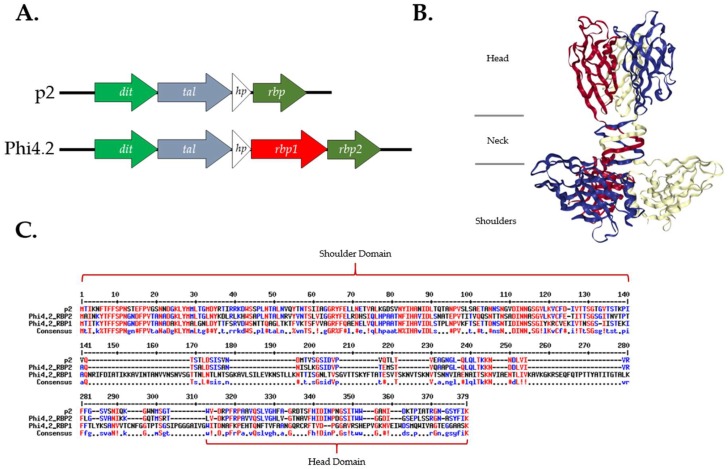
Sequence analysis of the RBPs of Phi4.2 and p2. (**A**) Genomic architecture of the baseplate regions of p2 and Phi4.2. (**B**) Ribbon structure of the RBP trimer of p2, as previously determined [14,23], highlighting the “shoulder”, “neck”, and “head” domains. Ribbon structure retrieved from the PDB database (PDB ID: 1ZRU). (**C**) Alignment of the amino acid sequences of the RBPs of Phi4.2 and p2, highlighting the elongated “neck” domain of RBP1_Phi4.2_. Conserved amino acids are in red, and partially conserved in blue. Alignment created using Multalin [43].

Phages Phi4.2, Phi4R15L, and Phi4R16L possess an atypical gene sequence in the baseplate region of their genome in relation to other phages of the 936 group, incorporating an additional, elongated RBP gene, *rbp1* (Figure 2A). Analysis of deduced amino acid sequences suggests that the second putative RBP gene, *rbp2*, encodes a typical 936 group RBP, which displays a high degree of homology to the entire sequence of the RBP of p2 (Figure 2C). Alignments also highlighted significant homology between the N terminus, or “shoulder” domain, of RBP1_Phi4.2_ and those of RBP2_Phi4.2_ and p2, indicating it likely attaches to the Dit protein in the assembled baseplate. Several conserved amino acids were also present in the C terminus, or “head” domain, of RBP1_Phi4.2_. However, RBP1_Phi4.2_ differed greatly in its “neck” region, possessing a significantly elongated sequence over 100 amino acids longer than those of RBP2_Phi4.2_ and p2.

### 3.2. RBP1_Phi4.2_ Is a Structural Protein and Part of the Mature Virion

**Figure 3:**
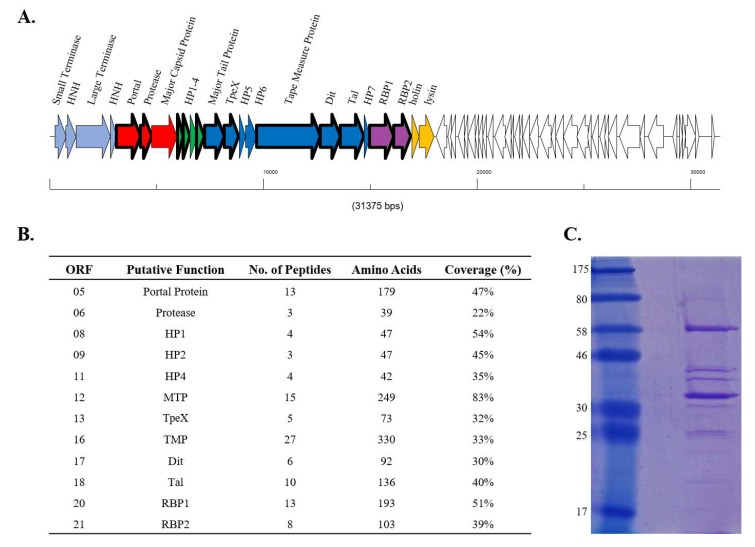
Summary of mass spectrometry data. (**A**) Schematic representation of the genetic architecture of Phi4.2. ORFs of the late region of the genome, which are known to code for the structural proteins of the phage, are represented in colour, with proteins detected by mass spectrometry reads bordered in bold. ORFs in light blue represent the packaging region of the genome, ORFs in red proteins of the phage head, ORFs in green unassigned hypothetical proteins, dark blue denotes genes encoding phage tail components, purple genes encoding the dual RBPs, and yellow represents genes involved in host lysis. (**B**) Summary of the peptide reads obtained from purified Phi4.2 particles via electrospray ionization-tandem mass spectrometry (ESI-MSI/MS). A minimum of either two independent unique peptides or 5% sequence coverage was used as threshold values. (**C**) The 12% SDS-PAGE of purified Phi4.2, from which bands were excised for ESI-MS/MS analysis. The ladder used is the 7–175 kDa broad-range protein ladder (NEB, Ipswich, MA, USA).

To determine whether both RBP1_Phi4.2_ and RBP2_Phi4.2_ are expressed and incorporated into the mature phage particle, proteins of the Phi4.2 virion were analysed via ESI-MS/MS. Most of the predicted tail-associated proteins of Phi4.2, such as the major tail protein, the tail tape measure protein, the TpeX (tail protein extension) protein, and the receptor binding protein, were detected in this analysis, although phage capsid proteins were not identified (a phenomenon previously observed in a number of studies, and likely a result of covalent cross-linking of head components resulting in oligomers too large to enter the gel [44,45]). A significant number of peptide reads were obtained for both RBP1_Phi4.2_ (13 unique reads covering 51% of the amino acid sequence) and RBP2_Phi4.2_ (8 unique reads covering 39% of the amino acid sequence), indicating both are indeed expressed and apparently incorporated into the phage particle (Figure 3B).

### 3.3. Phi4.2 Exhibits Distinct Features among 936 Group Phages

**Figure 4:**
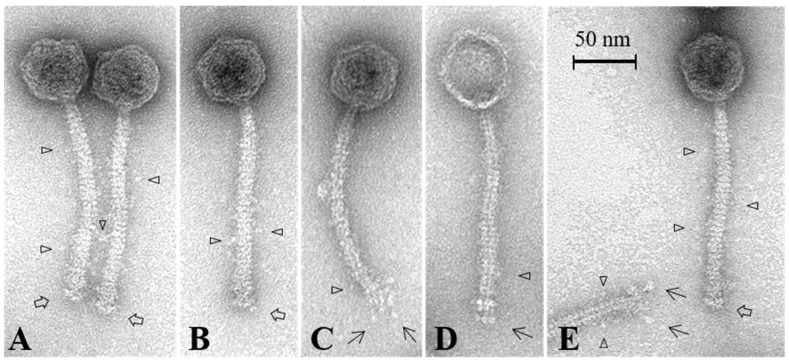
Representative micrographs of Phi4.2. **⇨** highlights the less compact baseplate of the phage. → highlights the elongated protein, thought to be RBP1_Phi4.2_, protruding from the baseplate of the phage (**C**,**D**,**E**). ▷ highlights the globular appendages which appear to coat the tail of the phage (**A**–**E**). Phages were stained using 2% uranyl acetate.

Phi4.2 was analysed by EM to determine if its structure differed from those of the archetypal 936 group phages. This analysis demonstrated that intact Phi4.2 phage particles possess an atypical baseplate (Figure 4A,B,E), which is significantly less compact than that of the previously analysed 936 phage p2 (Supplementary Figure S1). Micrographs of Phi4.2, as well as Phi4R15L and Phi4R16L, highlight that the phages possess an elongated baseplate when compared vertically with that of p2 (Supplementary Figure S1 and Table S2). While the baseplate of p2 measured 12.7 ± 1.3 nm in length, the baseplates of the Phi4.2-like phages measured 6–7 nm longer (Supplementary Table S2). Considering the very high sequence identity between the *dit*, *tal*, and *rbp/rbp2* genes in p2 and 4.2, this difference can likely be attributed to the presence of RBP1_Phi4.2_ in the baseplate. Comparative sequence analysis of RBP1_Phi4.2_ suggests a large insertion in the neck domain [14] which would likely cause the RBP1_Phi4.2_ head to protrude from the baseplate core. The RBP1_Phi4.2_ protein was visible as a drumstick-like appendage, mainly present in damaged phage particles where the staining solution had already penetrated into the central tail tube (Figure 4C,D,E), indicating distinct conformational changes of these distal complexes which exposed RBP1_Phi4.2_. Additionally, the phage tail appeared to be covered in a series of small, globular proteins, separated by roughly half a tail width from the tail itself, and possibly linked by fine, flexible fibres, although the resolution limits of the microscope made this difficult to discern (Figure 4A–E). These proteins may be a variation of the TpeX protein previously observed in 936 group proteins [5], with Phi4.2 previously noted as possessing a *tpeX* gene downstream of the *mtp gene* Phages Phi4R15L and Phi4R16L (which are also predicted to encode a dual RBP baseplate) demonstrated similar morphologies (Supplementary Figure S1), while imaging of p2 confirmed it did not possess these globular appendages on its tail (Supplementary Figure S1).

### 3.4. RBP1_Phi4.2_ Forms a Complex with Other Baseplate Components

Expression of a range of individual proteins and protein complexes was attempted in order to further characterise the baseplate-associated region of Phi4.2. Expression and purification of RBP2_Phi4.2_ (which demonstrates a higher similarity to other 936 group RBPs) was successful, with a high protein yield. Despite considerable efforts, RBP1_Phi4.2_ (which possesses an elongated “neck” region and is highly divergent from other 936 group RBPs) could not be expressed in a soluble manner irrespective of the expression system or host used. These included the *L. lactis* vectors pNZ8048 and pTX8048 (incorporating a thioredoxin fusion tag to improve solubility), and the *E. coli* vectors pQE30, pETM11, and pETM30 (which incorporates a Glutathione S-Transferase (GST) tag to promote solubility). However, when expressed in fusion with mCherry, a very small amount of soluble mCherry-RBP1_Phi4.2_ fusion protein (0.048 mg/L of culture) was purified. The Dit and Tal proteins were equally insoluble when expressed individually. Expression of mCherry-RBP2_Phi4.2_ fusion protein resulted in purification of a very high yield of stable protein (173 mg/L).

**Figure 5:**
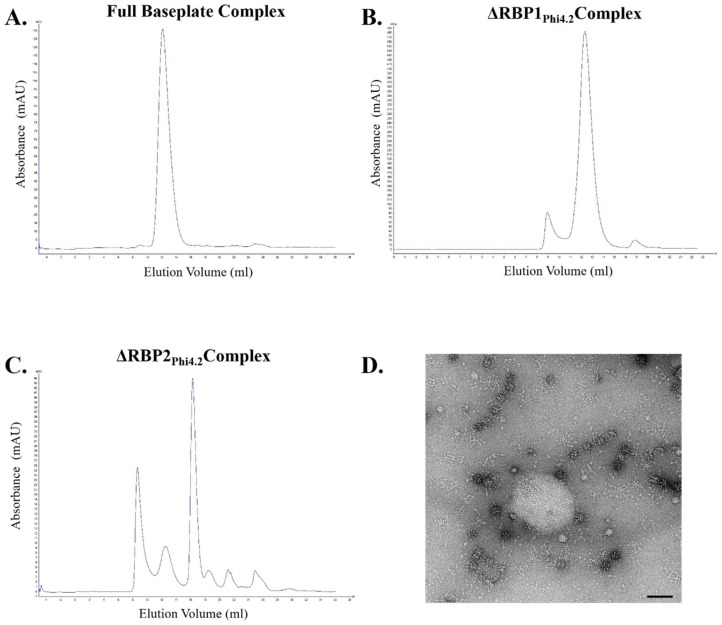
Gel filtration chromatograms of three Phi4.2-derived baseplate complexes analysing complex stability, with accompanying EM analysis of the ΔRBP2_Phi4.2_ complex: (**A**) analysis of the full baseplate; (**B**) analysis of the ΔRBP1_Phi4.2_ complex; (**C**) analysis of the ΔRBP2_Phi4.2_ complex; and (**D**) negative stain EM micrograph of the ΔRBP2_Phi4.2_ complex, highlighting its instability. In gel filtration chromatograms, the y-axis represents intensity of absorbance (milli Absorbance Units), and the x-axis represents the elution volume in millilitres. Protein complexes were observed using 1% uranyl-acetate staining and imaged on a FEI Tecnai Sphera LaB_6_ 200 kV microscope. Scale bars represent 100 nm. Gel filtration was performed using a Superose 6 Increase 10/300 GL column (GE Healthcare Life Sciences, Cork, Ireland), in a 50 mM Tris-HCl, 300 mM NaCl, pH 7.5 protein buffer.

Expression of the full baseplate region (encoding the Dit, Tal, HP, RBP1, and RBP2 proteins) resulted in the successful purification of the baseplate of Phi4.2, with a yield of 9 mg/L, and the purified complex was stable during assessment by gel filtration (Figure 5A). The ΔRBP1_Phi4.2_ complex, in which RBP1_Phi4.2_ was removed via SOEing PCR, expressed at higher levels with a yield of 16.2 mg/L and was also highly stable (Figure 5B). In contrast, expression of the ΔRBP2_Phi4.2_ complex produced a much lower yield of 2.75 mg/L of protein and was highly unstable, with the vast majority of the protein complex degraded after 24 h of storage at 4 °C (Figure 5C). Negative staining EM analysis of this complex further confirmed its instability (Figure 5D), with numerous disassembled components visible, including many drumstick-like structures possibly indicating dissociated RBP1_Phi4.2_. The ΔHP_Phi4.2_ construct, which lacks the small protein of unknown function, did not allow detection of protein expression in any examined fraction. The Dit_Phi4.2_ + Tal_Phi4.2_ complex (also incorporating the hypothetical protein) was not produced in the soluble fraction.

### 3.5. Both RBP1_Phi4.2_ and RBP2_Phi4.2_ Bind Specifically to Lactococcal Host Cells

**Figure 6:**
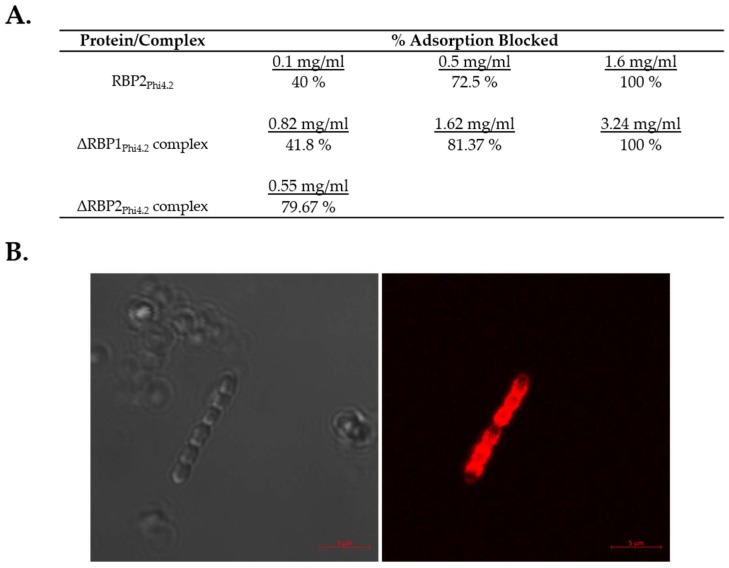
Adsorption inhibition and fluorescent binding assay results. (**A**) Adsorption inhibition assays involving the incubation of host *L. lactis* 4 with either RBP2_Phi4.2_, the ΔRBP1_Phi4.2_ complex, or the ΔRBP2_Phi4.2_ complex. Due to the instability of the ΔRBP2_Phi4.2_ complex, adsorption inhibition assays were performed using protein taken directly after gel filtration purification, resulting in only one concentration of protein being examined. All adsorption inhibition assays were performed in triplicate. (**B**) Fluorescent binding assays using mCherry tagged RBP1_Phi4.2_. Protein was added at a quantity of 5 µg/mL. Scale bars correspond to 5 µm. Cells were visualized using differential interference contrast (DIC) microscopy (panel on the left), and fluorescent confocal microscopy (panel on the right) at the mCherry excitement wavelength of 514 nm.

The proteins and protein complexes that were successfully expressed and purified were applied to adsorption inhibition assays to determine which proteins play a role in the binding of Phi4.2 to its host. For this purpose RBP2_Phi4.2_, the ΔRBP1_Phi4.2_ baseplate complex, and the ΔRBP2_Phi4.2_ baseplate complex were incubated with exponential-phase *L. lactis* 4 host cells at a variety of concentrations (with the exception of ΔRBP2_Phi4.2_, which was only examined at a single concentration using protein taken directly after gel filtration due to the instability of the complex). Incubation of *L. lactis* 4 with each of RBP2_Phi4.2_, the ΔRBP1_Phi4.2_ baseplate complex, and the ΔRBP2_Phi4.2_ baseplate complex resulted in significantly reduced phage adsorption in comparison to the control (which involved incubation of the cells with protein buffer, and determined base adsorption to be 46.8%). In the case of RBP2_Phi4.2_, a protein concentration of 1.6 mg/mL was shown to completely eliminate phage adsorption to the host, while 40% adsorption inhibition was observed at concentrations as low as 0.1 mg/mL (Figure 6A). Similarly, incubation with the ΔRBP1_Phi4.2_ complex had a major impact on phage adsorption, with adsorption being completely blocked at 3.24 mg/mL, confirming the role of RBP2_Phi4.2_ in host binding. Fluorescent binding assays using the purified mCherry-RBP2_Phi4.2_ fusion protein at a final concentration of 50 µg/mL resulted in strong labelling (Supplementary Figure S2), further confirming its role as a typical RBP. In the case of the ΔRBP2_Phi4.2_ complex, 80% of phage adsorption was blocked at a concentration of 0.55 mg/mL (Figure 6A). Additionally, fluorescent binding assays using the purified mCherry-RBP1_Phi4.2_ fusion protein were performed, and host binding was observed (Figure 6B), although at a very low level (roughly one in every 30–50 cells), possibly due to the minute quantity of protein employed (5 µg/mL), while no binding was observed in the case of the control strain *L. lactis* 10.Thus, RBP1_Phi4.2_ also appears to play a role in the host binding process.

### 3.6. Western Blot and Immunogold Labelling

**Figure 7:**
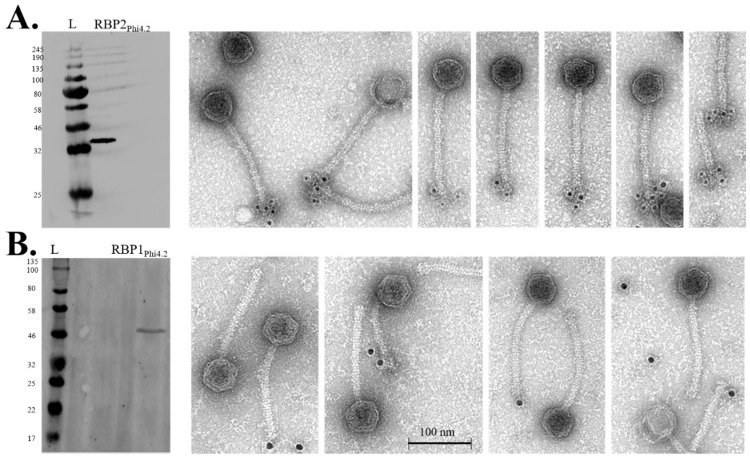
Western hybridisation and immunogold labelling of Phi4.2 using individual antibodies against RBP2_Phi4.2_ (raised against purified RBP2 protein), and RBP1_Phi4.2_ (raised against purified SDS-PAGE bands produced by the full baseplate complex). (**A**) Western hybridisation analysis of RBP2_Phi4.2_ (on the left), and immunogold labelling (on the right) using 5 nm gold conjugates (Sigma-Aldrich, Wicklow, Ireland). (**B**) Western hybridisation analysis of RBP1_Phi4.2_ (on the left), and immunogold labelling (on the right) using 10 nm gold conjugates (Sigma-Aldrich, Wicklow, Ireland). For all blots, the New England Biolabs Color Prestained Protein Standard, Broad Range (11–245 kDa) was used (indicated by lanes marked “L”, molecular weights indicated in kDa on the left).

Using antibodies raised against RBP1_Phi4.2_ and RBP2_Phi4.2_, the presence of both proteins in the purified phage samples was confirmed by Western blot analysis (Figure 7). Immunogold electron microscopy was attempted to confirm their location in the tail. Specific labelling of the tail tip region of the phage was obtained reliably with anti-RBP2_Phi4.2_ antibodies (Figure 7A). However, in the case of anti-RBP1_Phi4.2_ antibodies, specific labelling proved to be much less consistent (Figure 7B) despite attempts with a range of gold conjugates (2, 5, and 10 nm), although weak labelling of the tail tip region was occasionally observed.

### 3.7. Electron Microscopy Analysis of the Baseplate of Phi4.2

**Figure 8:**
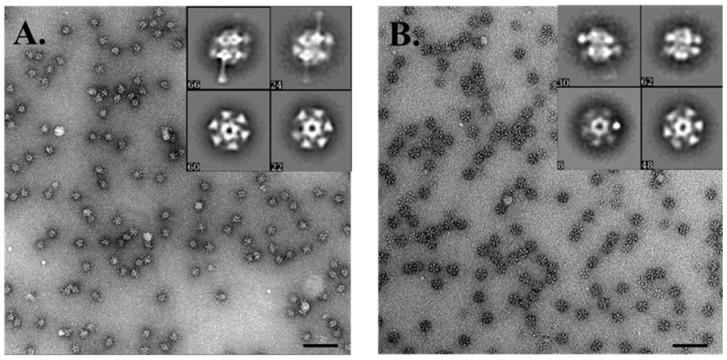
Electron microscopy analysis of the baseplate of Phi4.2: (**A**) negative stain imaging of the full baseplate of Phi4.2; and (**B**) negative stain analysis of the ΔRBP1Phi4.2 complex. (**A**,**B**) Scale bars correspond to 100 nm. Representative 2D classes of each complex are inset.

The purified full baseplate and ΔRBP1_Phi4.2_ protein complexes were analysed by negative staining electron microscopy in an attempt to obtain structural information on these heteromultimeric protein assemblies. During previous negative staining EM analysis of the 936 group phage p2 purified baseplate complex, the presence of two conformations was detected [13]: the closed, globular (or “heads-up”) form in the absence of Ca^2+^, and the open, star-like (or “heads-down”) form in the presence of Ca^2+^ and Sr^2+^. In the case of Phi4.2, we observed only the open, star-like conformation, likely due to the presence of Ca^2+^ in the protein buffer (Figure 8). Purification of the open form of the p2 baseplate resulted in a back-to-back dimerisation of pairs of individual baseplates. Lateral views of the open Phi4.2 baseplate and the ΔRBP1_Phi4.2_ complex revealed the presence of two “disks” of RBP heads, indicating both purified complexes have dimerised in this case as well, likely the result of a lack of a tail to attach to when expressed alone in *E. coli*. As both protein complexes proved unstable once Ca^2+^ was removed from the protein buffer, baseplates in the closed conformation could not be observed. Noteworthy, once the dimerisation is accounted for, both the full baseplate of Phi4.2 and the ΔRBP1_Phi4.2_ complex are comparable in structure to that of the open p2 baseplate, with the key difference being the presumed RBP1_Phi4.2_ protruding from the full Phi4.2 baseplate.

### 3.8. SEC/MALS Determination of Baseplate Size

Both the full baseplate and ΔRBP1Phi4.2 complexes were characterised via SEC/MALS/RI in order to determine their absolute mass (the instability of the ΔRBP2_Phi4.2_ complex precluded such an analysis). The measured molecular mass of the full baseplate was ~1.8 MDa (Supplementary Figure S3), roughly double the size of the previously characterised baseplate of the 936 phage p2 [13], a result which can largely be attributed to the dimerization mentioned previously. For the ΔRBP1_Phi4.2_ derived complex, a molar mass of ~1.4 MDa was obtained. In both cases, the accuracy of the molar mass could not be refined further due to the instability of the peak, likely a result of the instability of the complexes. The theoretical mass of two back-to-back open baseplates (each composed of 6 Dit proteins, 3 Tal proteins, and 18 RBPs) is 2.12 MDa if it was composed of only RBP1, and 1.7 MDa if it was composed of only RBP2. The observed mass of ~1.8 MDa indicates a mixture of RBP1 and RBP2 in the native phage 4.2 baseplate. The theoretical mass of back-to-back ΔRBP1Phi4.2 complex (incorporating two full complements of 18 RBP2_Phi4.2_ proteins) is 1.68 MDa, larger than the observed value of ~1.4 MDa, which can be explained by the absence of RBP1_Phi4.2_ in the baseplate. A slight instability was observed during SEC/MALS characterisation of the full baseplate, with a second, smaller peak observed at a similar size to that of the ΔRBP1_Phi4.2_ derived complex, indicating possible dissociation of RBP1_Phi4.2_. Slight asymmetry was also observed in the peak produced for the full baseplate complex presented in Figure 5, although due to the lower resolution of the produced chromatogram it is less apparent.

## 4. Discussion

Analysis of the genomes of all 115 available 936 group lactococcal phages reveals that the architecture of the baseplate of this phage group is highly conserved, with 112 phages possessing the same gene order. However, the three phages mentioned in this study (Phi4.2, PhiR15L, and PhiR16L) are currently the exceptions to this general architectural rule, as they possess a second RBP-encoding gene, *rbp1*, which exhibits an extended 3’ end and thus does not match previously determined groupings of 936 group phage RBPs. Through detailed characterisation of Phi4.2, the representative phage for this dual-RBP phenomenon, it was revealed that these exceptions possess a number of significant differences to the typical 936 phage structure.

EM analysis of the whole phage has revealed that the phage possesses an atypical baseplate, which is significantly less compact in comparison to the previously studied phage p2 [13]. Negative staining EM analysis of the full expressed baseplate and the ΔRBP1_Phi4.2_ complex indicates that each is in the “open” conformation, which resulted in dimerization in solution (an observation previously observed in the characterisation of the *B. subtilis* phage SPP1 [46], which also possesses a hexameric Dit conformation, and likely a result of the absence of its partners when expressed alone in *E. coli* [11]). Comparison of the two structures indicates that, in each, the Dit ring possesses a near-complete complement of 18 RBP2_Phi4.2_ proteins, with the full baseplate incorporating an undetermined, but much lower number of highly flexible RBP1_Phi4.2_ proteins protruding from the structure. The molecular mass of the back-to-back open baseplates as determined by SEC-MALS analysis supports this, with the determined mass of ~1.8 MDa indicating that the heteromultimeric protein complex may be composed of two Tal trimers (6 × 42.9 kDa), two Dit hexamers (12 × 34.4 kDa), and two near-complete compliments of 18 RBP2_Phi4.2_ proteins (36 × 28.6 kDa), giving a total of 1.69 MDa, with the difference accounted for by the incorporation of the larger RBP1_Phi4.2_ proteins (a full RBP1_Phi4.2_ baseplate dimer would be 2.12 MDa).

Purification of RBP1_Phi4.2_ alone was not achievable in this study due to its insolubility, likely a result of its elongated “neck” region, composed of difficult-to-fold interlaced beta-helix folds [14]. Furthermore, precise localisation of RBP1_Phi4.2_ was difficult due to the weak labelling observed during immunogold labelling. The weaker interactions of anti-RBP1_Phi4.2_ antibodies in comparison to those of RBP2_Phi4.2_ may be due to a number of reasons. For example, as the antibody was raised against denatured protein extracted from SDS-PAGE gel slices, it may not recognise the native, folded protein. Secondly, RBP1_Phi4.2_ may not be sufficiently exposed for labelling, with the drumstick-like protein primarily visible in damaged phage particles during EM analysis. Finally, as the polyclonal antibodies were raised against much lower quantities of protein than in the case of RBP2_Phi4.2_, they may simply possess a low affinity for RBP1_Phi4.2_. However, through fluorescent binding with an RBP1_Phi4.2_-mCherry fusion protein, and adsorption assays using the ΔRBP2_Phi4.2_ complex, RBP1_Phi4.2_ has been demonstrated to bind to the host, and therefore is highly likely to play a role in phage–host interactions. Numerous factors may be at play in relation to the low binding levels observed for RBP1_Phi4.2_-mCherry in comparison to RBP2_Phi4.2_. Low protein concentrations, and poor complex stability undoubtedly play a role. However, the host receptor for the RBP may also be a factor; while the head domains of both RBPs showed a degree of similarity, the sequence at amino acid level was quite different. Thus, it cannot be precluded that RBP1_Phi4.2_ binds a slightly different receptor to that of RBP2_Phi4.2_, which may be more exposed on the labelled cells.

These results raise the question as to why it would be advantageous for the phage to incorporate both of these RBPs. In fact, as demonstrated by the improved expression and greater stability of the ΔRBP1_Phi4.2_ complex in comparison to the full baseplate, it appears that the RBP1_Phi4.2_ protein is difficult to produce and incorporate into the baseplate structure. However, when viewing this phenomenon in the context of the host ranges of these phages, possible reasons for the incorporation of this second RBP emerge. These three phages were isolated from the same dairy facility against the same starter culture host (*L. lactis* 4) [4,5]. Interestingly, while all other phages isolated from these facilities demonstrated a relatively broad host range and all infected multiple strains, these three phages only infected *L. lactis* 4, and were also the only phages to do so. Thus, it appears that the dual-RBP phenomenon may be a specific adaptation to this host, allowing the phages to fill a niche in the starter culture. This is in apparent contrast to Twort-like phages infecting Gram-positive *Staphylococcus aureus*, where the presence of two RBPs contributes to a broad host range [47].

With the exception of the ΔRBP1_Phi4.2_ complex, the removal of any part of the baseplate results in either a lack of expression (ΔHP_Phi4.2_), insolubility (RBP1_Phi4.2_, Dit_Phi4.2_, and Tal_Phi4.2_ proteins individually), or a high degree of instability (ΔRBP2_Phi4.2_). This implies that assembly of this complex during phage propagation must occur rapidly to allow formation of a stable baseplate, as previously observed [13]. The lack of protein expression in the case of the ΔHP_Phi4.2_ construct indicates that it is required for baseplate assembly. However, the ΔHP_Phi4.2_ protein is not part of the final complex. Thus, this indicates that the *hp_Phi4.2_* gene encodes a chaperone protein required for baseplate assembly.

Anomalous globular appendages were observed coating the phage tail of Phi4.2, which were presumed to be encoded by the *tpeX* gene, as opposed to the smaller adhesin domains present on the tail of p2, which are encoded by the C-terminal end of the MTP [48]. However, these globular structures do not resemble previously described TpeX tail accessories observed in 936 group phages [5], despite possessing similar sequence lengths and amino acid sequence similarity between the Phi4.2 TpeX protein and comparable TpeX extensions. For example, the TpeX of Phi4.2 exhibited homology with that of PhiC0139 (99% coverage and 38% identity), with the vast majority of homology occurring in the N and C termini, where sequence identity increased to 78%. Preliminary HHpred analysis of the central portion of the protein sequence predicted structural homology with the BppA structure of the P335 phage Tuc2009 (PDB ID 5e7T), a baseplate component that encodes a Carbohydrate Binding Module (CBM) involved in host binding. Thus, the spiral-like structure apparent on the tail of PhiC0139 and the globular appendages visible on the tails of the three phages examined in this study, thought to be the TpeX fused to the MTP through a programmed +1 frameshift [5], highlight the structural diversity of these potential host-binding adaptations.

The flexibility of the globular tail appendages and the protruding RBP1_Phi4.2_ proteins ultimately resulted in Phi4.2 being recalcitrant to detailed structural characterisation. However, initial questions have been answered in this study of Phi4.2; both RBPs are produced and incorporated into the mature phage, and both appear to be involved in host–phage interactions. This places the three phages mentioned in this study as the only lactococcal 936 phages possessing two distinct RBPs which have been proven to be simultaneously incorporated into the mature phage particle, and to both be involved in host binding.

## 5. Conclusions

Phages of *L. lactis* remain a constant cause of economic loss in the dairy industry. Improving our understanding of the host-binding machinery of these phages and how they infect their hosts is an integral part of the design and improvement of strategies to limit their impact on industrial processes [49]. While the host binding machinery of 936 phage group appears to be relatively conserved, studies such as these highlight that variation and specific adaptations to fit biological niches do occur. The unique baseplate structure of the phages examined in this study emphasises the potential diversity and genetic elasticity of these phages, and highlights the adaptations still to be unearthed even amongst relatively well characterised phage groups.

## Supplementary Materials

The following are available online at http://www.mdpi.com/1999-4915/10/12/668/s1, Figure S1: EM analysis of phages p2, Phi4R15L and Phi4R16L, Figure S2: Fluorescent binding assays using mCherry tagged RBP2_Phi4.2_, Figure S3: SEC/MALS/RI analysis of the full baseplate and the ΔRBP1 complexes, Table S1: Oligonucleotides used in this study, Table S2: Measurements of morphological features of the examined phages.
