# Supplementary material for: Identification of Dual Receptor Binding Protein Systems in Lactococcal 936 Group Phages
Source: Viruses. 2018 Nov 27;10(12):668. doi: 10.3390/v10120668 (PMC6315561; doi:10.3390/v10120668)
Supplement: Supplementary file 1 [file viruses-10-00668-s001.pdf]

viruses

Supplementary Data

Identification of dual receptor binding protein systems in lactococcal 936 group phages

Stephen Hayes [1], Yoan Duhoo [2]; Horst Neve [3]; James Murphy [1]; Jean-Paul Noben [4], Charles M. Franz [3]; Christian Cambillau [2, 5]; Jennifer Mahony [1]; Arjen Nauta[6] and Douwe van Sinderen [1*]

[1] School of Microbiology & APC Microbiome Ireland, University College Cork, Western Road, Cork T12 YT20; stephen.hayes@umail.ucc.ie; j.mahony@ucc.ie; d.vansinderen@ucc.ie.
[2] Architecture et Fonction des Macromolécules Biologiques, Centre National de la Recherche Scientifique (CNRS), Campus de Luminy, Marseille, France. Yoan.Duhoo@afmb.univ-mrs.fr; cambillau@afmb.univ-mrs.fr
[3] Department of Microbiology and Biotechnology, Max Rubner-Institut, Kiel, Germany; horst.neve@mri.bund.de; charles.franz@mri.bund.de.
[4] Biomedical Research Institute, Hasselt University, Diepenbeek, Belgium
[5] Architecture et Fonction des Macromolécules Biologiques, Aix-Marseille Université, Campus de Luminy, Marseille, France
[6] FrieslandCampina, Amersfoort, Netherlands arjen.nauta@frieslandcampina.com
* Correspondence: d.vansinderen@ucc.ie; Tel.: +353-21-4901365

Supplementary Table S1. Measurements of morphological features of the examined phages.

	Head diameter	Tail length incl. bp	Tail width	(Total) bp length	(Total) bp width
**p2**	54.2 ± 1.9 (n=23)	160.6 ± 5.9 (n=23)	12.9 ± 0.7 (n=23)	12.7 ± 1.3 (n=23)	18.1 ± 1.0 (n=23)
**4.2**	55.4 ± 2.6 (n=19)	158.6 ± 4.0 (n=19)	12.9 ± 0.7 (n=19)	18.6 ± 1.6 (n=12)	17.0 ± 1.2 (n=12)
**4R15L**	55.4 ± 1.7 (n=17)	159.6 ± 5.0 (n=17)	12.8 ± 0.7 (n=17)	19.8 ± 1.4 (n=11)	17.0 ± 1.3 (n=11)
**4R16L**	54.0 ± 2.1 (n=20)	158.5 ± 5.6 (n=20)	12.3 ± 0.6 (n=20)	19.0 ± 2.3 (n=8)	17.2 ± 1.1 (n=8)

All measurements are presented in nm.

Supplementary Table S2. Oligonucleotides used in this study.

Oligonucleotide	Sequence	Target
RBP2F	agcagcccatggcacaccatcaccatcaccattcttctggtataaataaatatactttttcagtcc	Forward primer for cloning of *rbp2* in pNZ8048
RBP2R	agcagcaagcttttatttaataaagtagcttgcg	Reverse primer for cloning of *rbp2* in pNZ8048
RBP1F	agcagcccatggcgcaccatcaccatcaccattcttctggtataacaaaatatacgtttttagtcc	Forward primer for cloning of *rbp1* in pNZ8048
RBP1R	agcagctctagattacttgctagcagctcctccc	Reverse primer for cloning of *rbp1* in pNZ8048 and pTX8048
RBP1pTXF	agcagcggatccatgacgataacaaaatatacg	Forward primer for cloning of *rbp1* in pTX8048
RBP1pETMF	agcagcccatggcaataaataaatatactttttcagtc	Forward primer for cloning of *rbp1* in pETM11
RBP1pETMR	agcagcggatccttacttgctagcagctcctccc	Reverse primer for cloning of *rbp1* in pETM11
BpF	agcagcccatggaagggagggcgtaatgcaccatcaccatcaccattcagtaagacagtataaaat	Forward primer for cloning of the baseplate region in pETM11
BpR	aggaggggatccttatttaataaagtagcttgc	Reverse primer for cloning of the baseplate region in pETM11
ΔRBP2R	aggaggggatccttacttgctagcagctcctccc	Reverse primer for cloning of the ΔRBP2 construct in pETM11
ΔRBP1R1	gtatatttatttattgccatttacatatcttccttttctac	Internal reverse primer used for the construction of the ΔRBP1 construct via SOEing PCR

ΔRBP1F$_2$	tagaaaaggaagatatgtaaatggcaataaataaatatac	Internal forward primer used for the construction of the ΔRBP1 construct via SOEing PCR
ΔHPR$_1$	cgtatattttgttatcgtcattttatcctctattcccctccataaagg	Internal reverse primer used for the construction of the ΔHP construct via SOEing PCR
ΔHPF$_2$	cctttatggaggggaatagaggataaaatgacgataacaaaatatacg	Internal forward primer used for the construction of the ΔHP construct via SOEing PCR
DitR	aggaggggatccttaaataaaatcaactttctttttg	Reverse primer for the cloning of the *dit* gene in pETM11
DitTalHPR	aggaggggatccttacatatcttccttttctacaatttgagc	Reverse primer for the cloning of the Dit and Tal complex in pETM11
TalF	agcagcccatggaagggagggcgtaatgcaccatcaccatcaccatttggcagaatataatttatatg	Forward primer for cloning of the *tal* gene in pETM11
TalR	aggaggggatccttatcctctattcccctccata	Reverse primer for cloning of the *tal* gene in pETM11
pNZ8048F	caggagaagggacgatagca	Forward checking primer for pNZ8048 and pTX8048
pNZ8048R	tcttccttattctcgctttg	Reverse checking primer for pNZ8048 and pTX8048
pETM11F	gattacgacatccccactactg	Forward checking primer for pETM11 and pETM30
pETM11R	cgggctttgttagcagccggatc	Reverse checking primer for pETM11 and pETM30
pQE30F	cagggttattgtctcatgagcg	Forward checking primer for pQE30
pQE30R	cagctcaccgtctttcattgcc	Reverse checking primer for pQE30

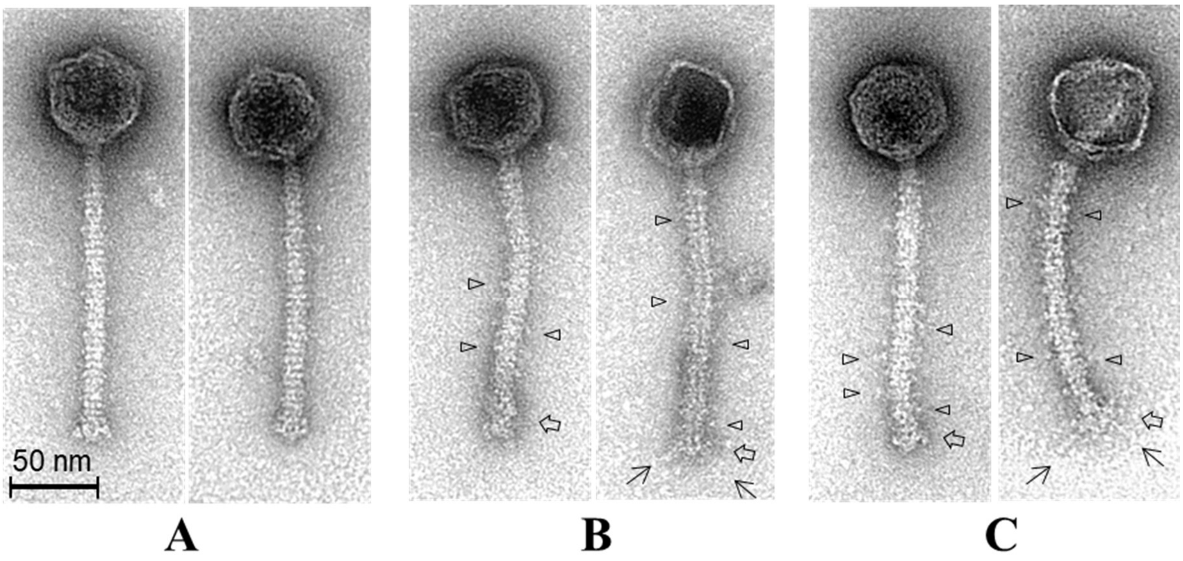

Figure S1. Representative micrographs of phages p2 (A), PhiR15L (B) and PhiR16L (C) stained with 2% uranyl acetate. ⇨ highlights the enlarged baseplate of phages PhiR15L (B) and PhiR16L (C). ▷ highlights some of the globular appendages which appear to coat the tail of the phages PhiR15L (B) and PhiR16L (C). PhiR15L and PhiR16L phage particles with empty heads (particles on right side in B & C) also show elongated appendages protruding from the baseplate (indicated with →).

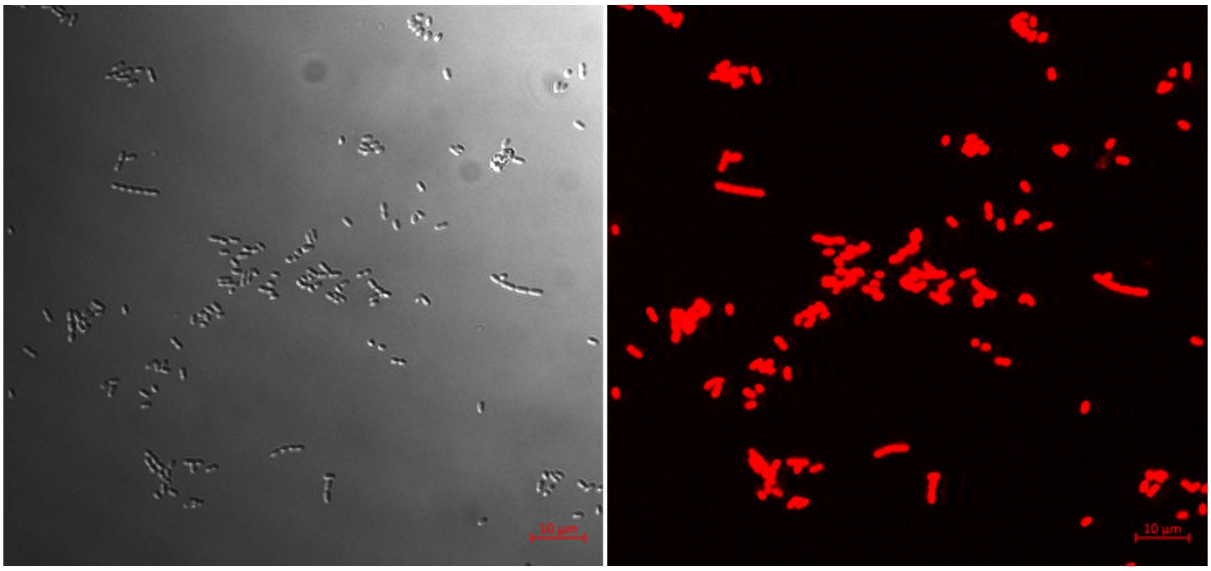

Figure S2. Fluorescent binding assays using mCherry tagged $RBP2_{Phi4.2}$. Protein was added at a concentration of 50 μg/ml. Scale bars correspond to 10 μm. Cells were visualized using differential interference contrast (DIC) microscopy (panel on the left), and fluorescent confocal microscopy (panel on the right) at the mCherry excitement wavelength of 514 nm.

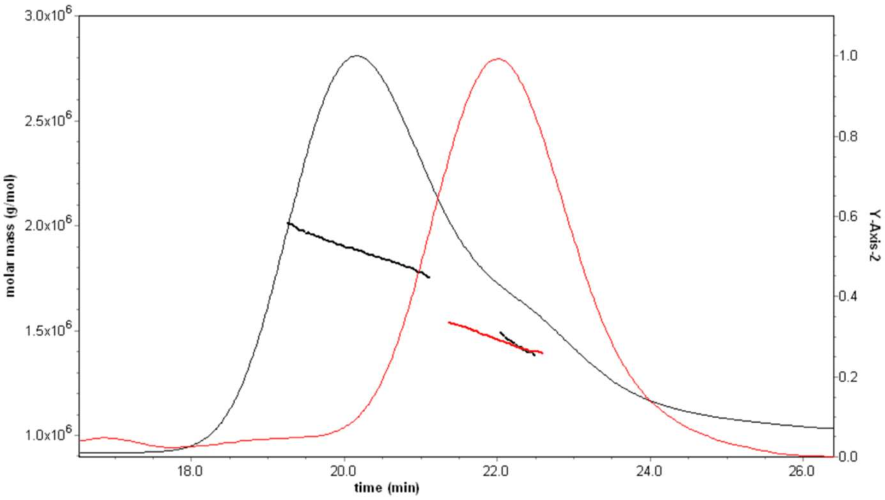

Figure S3. SEC/MALS/RI analysis of the full baseplate (black curve) and the ΔRBP1 (red curve) complexes. The molar mass (left axis), and the UV280nm absorbance (right axis) are plotted as a function of the column elution time. The column used was a 24-ml Superose 6 HR10/30 column (GE Healthcare, Cork, Ireland.
